# Supplementary material for: The membrane-associated proteins FCHo and SGIP are allosteric activators of the AP2 clathrin adaptor complex
Source: eLife. 2014 Oct 10;3:e03648. doi: 10.7554/eLife.03648 (PMC4215536; doi:10.7554/eLife.03648)
Supplement: Supplementary file 1. — Worm strains, plasmids, and oligonucleotide sequences. (A) Extended Strains List (B) Plasmids (C) Oligonucleotides. DOI: http://dx.doi.org/10.7554/eLife.03648.021 [file elife03648s001.doc]

**Supplementary file 1.** Worm strains, plasmids, and oligonucleotide sequences

**(A) Extended Strains List**

Wild-type

Bristol N2

*fcho-1* MosDEL

EG6353 *fcho-1(ox477::unc-119(+)) II ; unc-119(ed3) III*

jowls screen

EG7126 *apm-2(ox499[M1?]) X*

EG8523 *apm-2(ox546[W64X]) X*

EG7128 *apm-2(ox501[R174X]) X*

EG7129 *apa-2(ox502[splice site]) X*

EG7132 *apa-2(ox505[W255X]) X*

EG7134 *apa-2(ox507[Q747X]) X*

EG7130 *apa-2(ox503[Q838X]) X*

EG7127 *fcho-1(ox500[Q634X]) II*

EG7131 *fcho-1(ox504[frameshift]) II*

EG8521 *fcho-1(ox619[K782X]) II*

EG8522 *fcho-1(ox620[W882X]) II*

Independently identified jowls mutant

EG2711 *fcho-1(dx34) II*

*fcho-1* suppressor screen

EG8450 *fcho-1(ox477::unc-119(+)) II ; apm-2(ox548[V101K]) X*

EG8451 *fcho-1(ox477::unc-119(+)) II ; apm-2(ox549[E110K]) X*

EG8452 *fcho-1(ox477::unc-119(+)) II ; apm-2(ox550[T160P]) X*

EG8453 *fcho-1(ox477::unc-119(+)) II ; apm-2(ox551[T160I]) X*

EG8454 *fcho-1(ox477::unc-119(+)) II ; apm-2(ox552[T160A]) X*

EG8455 *fcho-1(ox477::unc-119(+)) II ; apm-2(ox553[H199Q]) X*

EG8456 *fcho-1(ox477::unc-119(+)) II ; apm-2(ox554[H199R]) X*

EG8457 *fcho-1(ox477::unc-119(+)) II ; apm-2(ox555[D248E]) X*

EG8458 *fcho-1(ox477::unc-119(+)) II ; apm-2(ox556[D248V]) X*

EG8459 *fcho-1(ox477::unc-119(+)) II ; apm-2(ox557[R292H]) X*

EG8460 *fcho-1(ox477::unc-119(+)) II ; apm-2(ox558[R292H]) X*

EG8461 *fcho-1(ox477::unc-119(+)) II ; apm-2(ox559[I294S]) X*

EG8462 *fcho-1(ox477::unc-119(+)) II ; apm-2(ox560[I294N]) X*

EG8463 *fcho-1(ox477::unc-119(+)) II ; apm-2(ox561[R302H]) X*

EG8464 *fcho-1(ox477::unc-119(+)) II ; apm-2(ox562[E306K]) X*

EG8465 *fcho-1(ox477::unc-119(+)) II ; apm-2(ox563[E306A]) X*

EG8466 *fcho-1(ox477::unc-119(+)) II ; apm-2(ox564[E306G]) X*

EG8467 *fcho-1(ox477::unc-119(+)) II ; apm-2(ox565[V310G]) X*

EG8468 *fcho-1(ox477::unc-119(+)) II ; apm-2(ox566[S371F]) X*

EG8469 *fcho-1(ox477::unc-119(+)) II ; apm-2(ox567[E373K]) X*

EG8470 *fcho-1(ox477::unc-119(+)) II ; apm-2(ox568[K384E]) X*

EG8471 *fcho-1(ox477::unc-119(+)) II ; apm-2(ox569[K385E]) X*

EG8472 *fcho-1(ox477::unc-119(+)) II ; apm-2(ox570[R389C]) X*

EG8473 *fcho-1(ox477::unc-119(+)) II ; apm-2(ox571[Y409N]) X*

EG8474 *fcho-1(ox477::unc-119(+)) II ; apm-2(ox572[R440S]) X*

EG8475 *fcho-1(ox477::unc-119(+)) II ; apa-2(ox573[H318R]) X*

EG8476 *fcho-1(ox477::unc-119(+)) II ; apa-2(ox574[L347F]) X*

EG8477 *fcho-1(ox477::unc-119(+)) II ; apa-2(ox575[M352R]) X*

EG8478 *fcho-1(ox477::unc-119(+)) II ; apa-2(ox576[Y393H]) X*

EG8479 *fcho-1(ox477::unc-119(+)) II ; apa-2(ox577[Y393H]) X*

EG8480 *fcho-1(ox477::unc-119(+)) II ; apa-2(ox578[C396W]) X*

EG8481 *fcho-1(ox477::unc-119(+)) II ; apa-2(ox579[L409F]) X*

EG8482 *fcho-1(ox477::unc-119(+)) II ; apa-2(ox580[L409F]) X*

EG8483 *fcho-1(ox477::unc-119(+)) II ; apa-2(ox581[L409F]) X*

EG8484 *fcho-1(ox477::unc-119(+)) II ; apa-2(ox582[M423K]) X*

EG8485 *fcho-1(ox477::unc-119(+)) II ; apa-2(ox583[M423R]) X*

EG8486 *fcho-1(ox477::unc-119(+)) II ; apa-2(ox584[M423K]) X*

EG8487 *fcho-1(ox477::unc-119(+)) II ; apa-2(ox585[Y441C]) X*

EG8488 *fcho-1(ox477::unc-119(+)) II ; apa-2(ox586[Y441C]) X*

EG8489 *fcho-1(ox477::unc-119(+)) II ; apa-2(ox587[R450H]) X*

EG8490 *fcho-1(ox477::unc-119(+)) II ; apa-2(ox588[R450H]) X*

EG8491 *fcho-1(ox477::unc-119(+)) II ; apa-2(ox589[G453D]) X*

EG8492 *fcho-1(ox477::unc-119(+)) II ; apa-2(ox590[E458K]) X*

EG8493 *fcho-1(ox477::unc-119(+)) II ; apa-2(ox591[E458K]) X*

EG8494 *fcho-1(ox477::unc-119(+)) II ; apa-2(ox592[N470I]) X*

EG8495 *fcho-1(ox477::unc-119(+)) II ; apa-2(ox593[A478T]) X*

EG8496 *fcho-1(ox477::unc-119(+)) II ; apa-2(ox594[T481A]) X*

EG8497 *fcho-1(ox477::unc-119(+)) II ; apa-2(ox595[T481A]) X*

EG8498 *fcho-1(ox477::unc-119(+)) II ; apa-2(ox596[T481P]) X*

EG8499 *fcho-1(ox477::unc-119(+)) II ; apa-2(ox597[T481A]) X*

EG8500 *fcho-1(ox477::unc-119(+)) II ; apb-1(ox598[T9I]) III*

EG8501 *fcho-1(ox477::unc-119(+)) II ; apb-1(ox599[E14G]) III*

EG8502 *fcho-1(ox477::unc-119(+)) II ; apb-1(ox600[I15R]) III*

EG8503 *fcho-1(ox477::unc-119(+)) II ; apb-1(ox601[F16S]) III*

EG8504 *fcho-1(ox477::unc-119(+)) II ; apb-1(ox602[Y325H]) III*

EG8505 *fcho-1(ox477::unc-119(+)) II ; apb-1(ox603[Y325C]) III*

EG8506 *fcho-1(ox477::unc-119(+)) II ; apb-1(ox604[A359V]) III*

EG8507 *fcho-1(ox477::unc-119(+)) II ; apb-1(ox605[A359V]) III*

EG8508 *fcho-1(ox477::unc-119(+)) II ; apb-1(ox606[E361K]) III*

EG8509 *fcho-1(ox477::unc-119(+)) II ; apb-1(ox607[D363G]) III*

EG8510 *fcho-1(ox477::unc-119(+)) II ; apb-1(ox608[D363A]) III*

EG8511 *fcho-1(ox477::unc-119(+)) II ; apb-1(ox609[L395F]) III*

EG8512 *fcho-1(ox477::unc-119(+)) II ; apb-1(ox610[L395F]) III*

EG8513 *fcho-1(ox477::unc-119(+)) II ; apb-1(ox611[N474K]) III*

EG8514 *fcho-1(ox477::unc-119(+)) II ; aps-2(ox612[E27K]) X*

EG8515 *fcho-1(ox477::unc-119(+)) II ; aps-2(ox613[E51V]) X*

EG8516 *fcho-1(ox477::unc-119(+)) II ; aps-2(ox614[D72A]) X*

EG8517 *fcho-1(ox477::unc-119(+)) II ; aps-2(ox615[H85R]) X*

EG8518 *fcho-1(ox477::unc-119(+)) II ; aps-2(ox616[V88I]) X*

EG8519 *fcho-1(ox477::unc-119(+)) II ; aps-2(ox617[D102G]) X*

Spontaneous *fcho-1* suppressor

EG6688 *fcho-1(ox477::unc-119(+)) II oxSi108[Paps-2::APS-2:GFP unc-119(+)] II ; apm-2(ox618[R440S]) X*

Single-copy transgenes (MosSCIs)

AP2-alpha-GFP

EG8012 *oxSi254[Pdpy-30::APA-2::GFP unc-119(+)] II ; unc-119(ed3) III*

YxxΦ cargo

EG8608 *oxSi484[Pvha-6::GFP:CD4:YASV unc-119(+)] II ; unc-119(ed3) III*

Heatshock TEV protease

EG8531 *oxSi883[Phsp-16.41::TEV(protease) unc-119(+)] II ; unc-119(ed3) III*

AP2-mu2-tev-site (FLAG)

EG8525 *unc-119(ed3) III ; oxSi877[Papm-2::3xFLAG:APM-2:tev-site unc-119(+)] X*

AP2-mu2-tev-site (HA)

EG8524 *unc-119(ed3) III ; oxSi876[Papm-2::HA:APM-2:tev-site unc-119(+)] X*

EG8526 *unc-119(ed3) III ; oxSi878[Papm-2::HA:APM-2(T160A):tev-site unc-119(+)] X*

EG8527 *unc-119(ed3) III ; oxSi879[Papm-2::HA:APM-2(T160E):tev-site unc-119(+)] X*

EG8528 *unc-119(ed3) III ; oxSi880[Papm-2::HA:APM-2(E306K):tev-site unc-119(+)] X*

EG8529 *unc-119(ed3) III ; oxSi881[Papm-2::HA:APM-2(K411E):tev-site unc-119(+)] X*

EG8530 *unc-119(ed3) III ; oxSi882[Papm-2::HA:APM-2(R440S):tev-site X*

FCHO-1 structure/function

EG7144 *unc-119(ed3) III ; oxSi556[Pdpy-30::HA:tagRFP:Ce_FCHO-1(1-968) unc-119(+)] IV*

EG8532 *unc-119(ed3) III ; oxSi884[Pdpy-30::HA:tagRFP:Ce_FCHO-1(288-968) unc-119(+)] IV*

EG7143 *unc-119(ed3) III ; oxSi555[Pdpy-30::HA:tagRFP:Ce_FCHO-1(536-968) unc-119(+)] IV*

EG8533 *unc-119(ed3) III ; oxSi885[Pdpy-30::HA:tagRFP:Ce_FCHO-1(1-687) unc-119(+)] IV*

EG8534 *unc-119(ed3) III ; oxSi886[Pdpy-30::HA:tagRFP:Ce_FCHO-1(1-286) unc-119(+)] IV*

FCHo APA domains

EG8535 *unc-119(ed3) III ; oxSi887[Pdpy-30::HA:tagRFP:Ce_FCHO-1(454-565) Cb_unc-119(+)] IV*

EG8536 *unc-119(ed3) III ; oxSi888[Pdpy-30::HA:tagRFP:Mm_FCHo2(306-394) unc-119(+)] IV*

EG8537 *unc-119(ed3) III ; oxSi889[Pdpy-30::HA:tagRFP:Mm_FCHo1(304-393) unc-119(+)] IV*

EG8538 *unc-119(ed3) III ; oxSi890[Pdpy-30::HA:tagRFP:Mm_SGIP1(97-184) unc-119(+)] IV*

EG8539 *unc-119(ed3) III ; oxSi891[Pdpy-30::HA:tagRFP:Hs_FCHo1(305-402) unc-119(+)] IV*

Figure 1

AP2-alpha-GFP (coelomocyte imaging)

EG8012 fcho(+), see MosSCIs

EG6650 *fcho-1(ox477::unc-119(+)) II oxSi254[Pdpy-30::APA-2::GFP unc-119(+)] II*

Cargo assay

EG8608 fcho(+), see MosSCIs

EG8540 *fcho-1(ox477::unc-119(+)) II oxSi484[Pvha-6::GFP:CD4:YASV unc-119(+)] II*

Figure 1-figure supplement 1D

Cargo assay

EG8608 WT, EG8540 *fcho-1*, see Figure 1 cargo assay

EG8802 *oxSi484[Pvha-6::eGFP::CD4-YASV unc-119(+)] II ; apa-2(ox422) X*

EG8801 *fcho-1(ox477::unc-119(+)) II oxSi484[Pvha-6::GFP:CD4:YASV unc-119(+)] II ; apa-2(ox422) X*

EG8803 *oxSi484[Pvha-6::GFP:CD4:YASV unc-119(+)] II ; aps-2(tm2912) X*

EG8804 *fcho-1(ox477::unc-119(+)) II oxSi484[Pvha-6::GFP:CD4:YASV unc-119(+)] II ; aps-2(tm2912) X*

EG8805 *oxSi484[Pvha-6::GFP:CD4:YASV unc-119(+)] II ; apm-2(ox546[W64X]) X*

EG8806 *fcho-1(ox477::unc-119(+)) II oxSi484[Pvha-6::GFP:CD4:YASV unc-119(+)] II ; apm-2(ox546[W64X]) X*

Figure 2-figure supplement 1

Starvation assay

N2 Bristol fcho(+), see wild-type

EG6353 fcho(-), see *fcho-1* MosDEL

EG8450 (μV101K), EG8454 (μT160A), EG8456 (μH199R), EG8457 (μD248E), EG8458 (μD248V), EG8460 (μR292H), EG8469 (μE373K), EG8470 (μK384E), EG8516 (σD72A), EG8518 (σV88I), EG8519 (σD102G), EG8502 (βI15R), EG8506 (βA359V), EG8507 (βA359V), EG8508 (βE361K), EG8513 (βN474K), EG8479 (αY393H), EG8480 (αC396W), EG8482 (αL409F), EG8484 (αM423K), EG8485 (αM423R), EG8488 (αY441C), EG8489 (αR450H), EG8491 (αG453D), EG8492 (αE458K), EG8493 (αE458K), EG8494 (αN470I), EG8495 (αA478T), EG8498 (αT481P), see *fcho-1* suppressor screen

Cargo assay

EG8608 fcho(+), EG8540 fcho(-), see Figure 1 cargo assay

EG8541 *fcho-1(ox477::unc-119(+)) II oxSi484[Pvha-6::GFP:CD4:YASV unc-119(+)] II ; apm-2(ox551[T160I]) X*

EG8542 *fcho-1(ox477::unc-119(+)) II oxSi484[Pvha-6::GFP:CD4:YASV unc-119(+)] II ; apm-2(ox563[E306A]) X*

EG8543 *fcho-1(ox477::unc-119(+)) II oxSi484[Pvha-6::GFP:CD4:YASV unc-119(+)] II ; apm-2(ox566[S371F]) X*

EG8544 *fcho-1(ox477::unc-119(+)) II oxSi484[Pvha-6::GFP:CD4:YASV unc-119(+)] II ; apm-2(ox572[R440S]) X*

EG8545 *fcho-1(ox477::unc-119(+)) II oxSi484[Pvha-6::GFP:CD4:YASV unc-119(+)] II ; apa-2(ox580[L409F]) X*

EG8546 *fcho-1(ox477::unc-119(+)) II oxSi484[Pvha-6::GFP:CD4:YASV unc-119(+)] II ; apm-2(ox558[R292H]) X*

EG8547 *fcho-1(ox477::unc-119(+)) II oxSi484[Pvha-6::GFP:CD4:YASV unc-119(+)] II ; apm-2(ox567[E373K]) X*

EG8548 *fcho-1(ox477::unc-119(+)) II oxSi484[Pvha-6::GFP:CD4:YASV unc-119(+)] II ; apm-2(ox568[K384E]) X*

EG8549 *fcho-1(ox477::unc-119(+)) II oxSi484[Pvha-6::GFP:CD4:YASV unc-119(+)] II ; apm-2(ox569[K385E]) X*

EG8550 *fcho-1(ox477::unc-119(+)) II oxSi484[Pvha-6::GFP:CD4:YASV unc-119(+)] II ; aps-2(ox614[D72A]) X*

EG8551 *fcho-1(ox477::unc-119(+)) II oxSi484[Pvha-6::GFP:CD4:YASV unc-119(+)] II ; apa-2(ox573[H318R]) X*

EG8552 *fcho-1(ox477::unc-119(+)) II oxSi484[Pvha-6::GFP:CD4:YASV unc-119(+)] II ; apa-2(ox597[T481A]) X*

FRAP

EG8012 fcho(+), EG6650 fcho(-), see Figure 1 AP2-alpha-GFP

EG8553 *fcho-1(ox477::unc-119(+)) II oxSi254[Pdpy-30::APA-2::GFP unc-119(+)] II ; apm-2(ox572[R440S]) X*

EG8554 *fcho-1(ox477::unc-119(+)) II oxSi254[Pdpy-30::APA-2::GFP unc-119(+)] II ; apm-2(ox563[E306A]) X*

Figure 3

EG8555 *oxSi883[Phsp-16.41::TEV(protease) unc-119(+)] II ; apm-2(ox546[W64X]) X oxSi877[Papm-2::3xFLAG:APM-2:tev-site unc-119(+)] X*

EG8556 *fcho-1(ox477::unc-119(+)) II oxSi883[Phsp-16.41::TEV(protease) unc-119(+)] II ; apm-2(ox546[W64X]) X oxSi877[Papm-2::3xFLAG:APM-2:tev-site unc-119(+)] X*

Figure 3-figure supplement 1

EG8557 *oxSi883[Phsp-16.41::TEV(protease) unc-119(+)] II ; apm-2(ox546[W64X]) X oxSi876[Papm-2::HA:APM-2:tev-site unc-119(+)] X*

EG8558 *fcho-1(ox477::unc-119(+)) II oxSi883[Phsp-16.41::TEV(protease) unc-119(+)] II ; apm-2(ox546[W64X]) X oxSi876[Papm-2::HA:APM-2:tev-site unc-119(+)] X*

Figure 4

Starvation, protease, and phosphorylation assays

EG8557 fcho(+), EG8558 fcho(-), see Figure 3-figure supplement 1

EG8559 *fcho-1(ox477::unc-119(+)) II oxSi883[Phsp-16.41::TEV(protease) unc-119(+)] II ; apb-1(ox603[Y325C]) III ; apm-2(ox546[W64X]) X oxSi876[Papm-2::HA:APM-2:tev-site unc-119(+)] X*

EG8560 *fcho-1(ox477::unc-119(+)) II oxSi883[Phsp-16.41::TEV(protease) unc-119(+)] II ; apb-1(ox608[D363A]) III ; apm-2(ox546[W64X]) X oxSi876[Papm-2::HA:APM-2:tev-site unc-119(+)] X*

EG8561 *fcho-1(ox477::unc-119(+)) II oxSi883[Phsp-16.41::TEV(protease) unc-119(+)] II ; apm-2(ox546[W64X]) X apa-2(ox573[H318R]) X oxSi876[Papm-2::HA:APM-2:tev-site unc-119(+)] X*

EG8562 *fcho-1(ox477::unc-119(+)) II oxSi883[Phsp-16.41::TEV(protease) unc-119(+)] II ; apm-2(ox546[W64X]) X apa-2(ox596[T481P]) X oxSi876[Papm-2::HA:APM-2:tev-site unc-119(+)] X*

EG8563 *fcho-1(ox477::unc-119(+)) II oxSi883[Phsp-16.41::TEV(protease) unc-119(+)] II ; apm-2(ox546[W64X]) X oxSi882[Papm-2::HA:APM-2(R440S):tev-site X*

EG8564 *fcho-1(ox477::unc-119(+)) II oxSi883[Phsp-16.41::TEV(protease) unc-119(+)] II ; apm-2(ox546[W64X]) X oxSi880[Papm-2::HA:APM-2(E306K):tev-site unc-119(+)] X*

EG8565 *fcho-1(ox477::unc-119(+)) II oxSi883[Phsp-16.41::TEV(protease) unc-119(+)] II ; apm-2(ox546[W64X]) X oxSi878[Papm-2::HA:APM-2(T160A):tev-site unc-119(+)] X*

EG8566 *fcho-1(ox477::unc-119(+)) II oxSi883[Phsp-16.41::TEV(protease) unc-119(+)] II ; apm-2(ox546[W64X]) X oxSi879[Papm-2::HA:APM-2(T160E):tev-site unc-119(+)] X*

Cargo assay

EG8608 fcho(+), EG8540 fcho(-), see Figure 1 cargo assay

EG8567 *fcho-1(ox477::unc-119(+)) II oxSi484[Pvha-6::GFP:CD4:YASV unc-119(+)] II ; apb-1(ox603[Y325C]) III*

EG8568 *fcho-1(ox477::unc-119(+)) II oxSi484[Pvha-6::GFP:CD4:YASV unc-119(+)] II ; apb-1(ox608[D363A]) III*

EG8569 *fcho-1(ox477::unc-119(+)) II oxSi484[Pvha-6::GFP:CD4:YASV unc-119(+)] II ; apa-2(ox573[H318R]) X*

EG8570 *fcho-1(ox477::unc-119(+)) II oxSi484[Pvha-6::GFP:CD4:YASV unc-119(+)] II ; apa-2(ox596[T481P]) X*

EG8571 *fcho-1(ox477::unc-119(+)) II oxSi484[Pvha-6::GFP:CD4:YASV unc-119(+)] II ; apm-2(ox546[W64X]) X oxSi882[Papm-2::HA:APM-2(R440S):tev-site X*

EG8572 *fcho-1(ox477::unc-119(+)) II oxSi484[Pvha-6::GFP:CD4:YASV unc-119(+)] II ; apm-2(ox546[W64X]) X oxSi880[Papm-2::HA:APM-2(E306K):tev-site unc-119(+)] X*

EG8573 *fcho-1(ox477::unc-119(+)) II oxSi484[Pvha-6::GFP:CD4:YASV unc-119(+)] II ; apm-2(ox546[W64X]) X oxSi878[Papm-2::HA:APM-2(T160A):tev-site unc-119(+)] X*

EG8574 *fcho-1(ox477::unc-119(+)) II oxSi484[Pvha-6::GFP:CD4:YASV unc-119(+)] II ; apm-2(ox546[W64X]) X oxSi879[Papm-2::HA:APM-2(T160E):tev-site unc-119(+)] X*

Figure 5

Starvation, protease, and phosphorylation assays

EG8557 fcho(+), EG8558 fcho(-), see Figure 3-figure supplement 1

EG8575 *fcho-1(ox477::unc-119(+)) II oxSi883[Phsp-16.41::TEV(protease) unc-119(+)] II ; apb-1(ox606[E361K]) III ; apm-2(ox546[W64X]) X oxSi876[Papm-2::HA:APM-2:tev-site unc-119(+)] X*

EG8576 *fcho-1(ox477::unc-119(+)) II oxSi883[Phsp-16.41::TEV(protease) unc-119(+)] II ; apm-2(ox546[W64X]) X oxSi881[Papm-2::HA:APM-2(K411E):tev-site unc-119(+)] X*

EG8577 *fcho-1(ox477::unc-119(+)) II oxSi883[Phsp-16.41::TEV(protease) unc-119(+)] II ; apb-1(ox606[E361K]) III ; apm-2(ox546[W64X]) X oxSi881[Papm-2::HA:APM-2(K411E):tev-site unc-119(+)] X*

Cargo assay

EG8578 *oxSi484[Pvha-6::GFP:CD4:YASV unc-119(+)] II ; apm-2(ox546[W64X]) X oxSi876[Papm-2::HA:APM-2:tev-site unc-119(+)] X*

EG8579 *fcho-1(ox477::unc-119(+)) II oxSi484[Pvha-6::GFP:CD4:YASV unc-119(+)] II ; apm-2(ox546[W64X]) X oxSi876[Papm-2::HA:APM-2:tev-site unc-119(+)] X*

EG8580 *fcho-1(ox477::unc-119(+)) II oxSi484[Pvha-6::GFP:CD4:YASV unc-119(+)] II ; apb-1(ox606[E361K]) III ; apm-2(ox546[W64X]) X oxSi876[Papm-2::HA:APM-2:tev-site unc-119(+)] X*

EG8581 *fcho-1(ox477::unc-119(+)) II oxSi484[Pvha-6::GFP:CD4:YASV unc-119(+)] II ; apm-2(ox546[W64X]) X oxSi881[Papm-2::HA:APM-2(K411E):tev-site unc-119(+)] X*

EG8582 *fcho-1(ox477::unc-119(+)) II oxSi484[Pvha-6::GFP:CD4:YASV unc-119(+)] II ; apb-1(ox606[E361K]) III ; apm-2(ox546[W64X]) X oxSi881[Papm-2::HA:APM-2(K411E):tev-site unc-119(+)] X*

Figure 6B

Structure/function (starvation and protease assays)

EG8555 fcho(+), EG8556 fcho(-), see Figure 3

EG8583 *fcho-1(ox477::unc-119(+)) II oxSi883[Phsp-16.41::TEV(protease) unc-119(+)] II ; oxSi556[Pdpy-30::HA:tagRFP:Ce_FCHO-1(1-968) unc-119(+)] IV ; apm-2(ox546[W64X]) X oxSi877[Papm-2::3xFLAG:APM-2:tev-site unc-119(+)] X*

EG8584 *fcho-1(ox477::unc-119(+)) II oxSi883[Phsp-16.41::TEV(protease) unc-119(+)] II ; oxSi884[Pdpy-30::HA:tagRFP:Ce_FCHO-1(288-968) unc-119(+)] IV ; apm-2(ox546[W64X]) X oxSi877[Papm-2::3xFLAG:APM-2:tev-site unc-119(+)] X*

EG8585 *fcho-1(ox477::unc-119(+)) II oxSi883[Phsp-16.41::TEV(protease) unc-119(+)] II ; oxSi555[Pdpy-30::HA:tagRFP:Ce_FCHO-1(536-968) unc-119(+)] IV ; apm-2(ox546[W64X]) X oxSi877[Papm-2::3xFLAG:APM-2:tev-site unc-119(+)] X*

EG8586 *fcho-1(ox477::unc-119(+)) II oxSi883[Phsp-16.41::TEV(protease) unc-119(+)] II ; oxSi885[Pdpy-30::HA:tagRFP:Ce_FCHO-1(1-687) unc-119(+)] IV ; apm-2(ox546[W64X]) X oxSi877[Papm-2::3xFLAG:APM-2:tev-site unc-119(+)] X*

EG8587 *fcho-1(ox477::unc-119(+)) II oxSi883[Phsp-16.41::TEV(protease) unc-119(+)] II ; oxSi886[Pdpy-30::HA:tagRFP:Ce_FCHO-1(1-286) unc-119(+)] IV ; apm-2(ox546[W64X]) X oxSi877[Papm-2::3xFLAG:APM-2:tev-site unc-119(+)] X*

Structure/function (cargo assay)

EG8588 *fcho-1(ox477::unc-119(+)) II oxSi484[Pvha-6::GFP:CD4:YASV unc-119(+)] II ; oxSi556[Pdpy-30::HA:tagRFP:Ce_FCHO-1(1-968) unc-119(+)] IV*

EG8589 *fcho-1(ox477::unc-119(+)) II oxSi484[Pvha-6::GFP:CD4:YASV unc-119(+)] II ; oxSi884[Pdpy-30::HA:tagRFP:Ce_FCHO-1(288-968) unc-119(+)] IV*

EG8590 *fcho-1(ox477::unc-119(+)) II oxSi484[Pvha-6::GFP:CD4:YASV unc-119(+)] II ; oxSi555[Pdpy-30::HA:tagRFP:Ce_FCHO-1(536-968) unc-119(+)] IV*

EG8591 *fcho-1(ox477::unc-119(+)) II oxSi484[Pvha-6::GFP:CD4:YASV unc-119(+)] II ; oxSi885[Pdpy-30::HA:tagRFP:Ce_FCHO-1(1-687) unc-119(+)] IV*

EG8592 *fcho-1(ox477::unc-119(+)) II oxSi484[Pvha-6::GFP:CD4:YASV unc-119(+)] II ; oxSi886[Pdpy-30::HA:tagRFP:Ce_FCHO-1(1-286) unc-119(+)] IV*

Figure 6C

APA extra-chromosomal arrays (starvation and protease assays)

EG8555 fcho(+), EG8556 fcho(-), see Figure 3

EG8593 *fcho-1(ox477::unc-119(+)) II oxSi883[Phsp-16.41::TEV(protease) unc-119(+)] II ; apm-2(ox546[W64X]) X oxSi877[Papm-2::3xFLAG:APM-2:tev-site unc-119(+)] X ; oxEx2028[Pdpy-30::HA:tagRFP:Ce_FCHO-1(454-565) unc-119(+)]*

EG8594 *fcho-1(ox477::unc-119(+)) II oxSi883[Phsp-16.41::TEV(protease) unc-119(+)] II ; apm-2(ox546[W64X]) X oxSi877[Papm-2::3xFLAG:APM-2:tev-site unc-119(+)] X ; oxEx2029[Pdpy-30::HA:tagRFP:Mm_FCHo2(306-394) unc-119(+)]*

EG8595 *fcho-1(ox477::unc-119(+)) II oxSi883[Phsp-16.41::TEV(protease) unc-119(+)] II ; apm-2(ox546[W64X]) X oxSi877[Papm-2::3xFLAG:APM-2:tev-site unc-119(+)] X ; oxEx2030[Pdpy-30::HA:tagRFP:Mm_SGIP1(97-184) unc-119(+)]*

EG8596 *fcho-1(ox477::unc-119(+)) II oxSi883[Phsp-16.41::TEV(protease) unc-119(+)] II ; apm-2(ox546[W64X]) X oxSi877[Papm-2::3xFLAG:APM-2:tev-site unc-119(+)] X ; oxEx2031[Pdpy-30::HA:tagRFP:Hs_FCHo1(305-402) unc-119(+)]*

APA extra-chromosomal arrays (cargo assay)

EG8608 fcho(+), EG8540 fcho(-), see Figure 1 cargo assay

EG8597 *fcho-1(ox477::unc-119(+)) II oxSi484[Pvha-6::GFP:CD4:YASV unc-119(+)] II ; oxEx2028[Pdpy-30::HA:tagRFP:Ce_FCHO-1(454-565) unc-119(+)]*

EG8598 *fcho-1(ox477::unc-119(+)) II oxSi484[Pvha-6::GFP:CD4:YASV unc-119(+)] II ; oxEx2029[Pdpy-30::HA:tagRFP:Mm_FCHo2(306-394) unc-119(+)]*

EG8599 *fcho-1(ox477::unc-119(+)) II oxSi484[Pvha-6::GFP:CD4:YASV unc-119(+)] II ; oxEx2030[Pdpy-30::HA:tagRFP:Mm_SGIP1(97-184) unc-119(+)]*

EG8600 *fcho-1(ox477::unc-119(+)) II oxSi484[Pvha-6::GFP:CD4:YASV unc-119(+)] II ; oxEx2031[Pdpy-30::HA:tagRFP:Hs_FCHo1(305-402) unc-119(+)]*

Figure 6-figure supplement 1

EG8012 fcho(+), EG6650 fcho(-), see Figure 1 AP2-alpha-GFP

EG8601 *fcho-1(ox477::unc-119(+)) II oxSi254[Pdpy-30::APA-2::GFP unc-119(+)] II ; oxSi884[Pdpy-30::HA:tagRFP:Ce_FCHO-1(288-968) unc-119(+)] IV*

EG8602 *fcho-1(ox477::unc-119(+)) II oxSi254[Pdpy-30::APA-2::GFP unc-119(+)] II ; oxEx2028[Pdpy-30::HA:tagRFP:Ce_FCHO-1(454-565) unc-119(+)]*

Figure 7-figure supplement 1A

APA single-copy transgenes (starvation assay)

EG8555 fcho(+), EG8556 fcho(-), see Figure 3

EG8603 *fcho-1(ox477::unc-119(+)) II oxSi883[Phsp-16.41::TEV(protease) unc-119(+)] II ; oxSi887[Pdpy-30::HA:tagRFP:Ce_FCHO-1(454-565) unc-119(+)] IV ; apm-2(ox546[W64X]) X oxSi877[Papm-2::3xFLAG:APM-2:tev-site unc-119(+)] X*

EG8604 *fcho-1(ox477::unc-119(+)) II oxSi883[Phsp-16.41::TEV(protease) unc-119(+)] II ; oxSi888[Pdpy-30::HA:tagRFP:Mm_FCHo2(306-394) unc-119(+)] IV ; apm-2(ox546[W64X]) X oxSi877[Papm-2::3xFLAG:APM-2:tev-site unc-119(+)] X*

EG8605 *fcho-1(ox477::unc-119(+)) II oxSi883[Phsp-16.41::TEV(protease) unc-119(+)] II ; oxSi889[Pdpy-30::HA:tagRFP:Mm_FCHo1(304-393) unc-119(+)] IV ; apm-2(ox546[W64X]) X oxSi877[Papm-2::3xFLAG:APM-2:tev-site unc-119(+)] X*

EG8606 *fcho-1(ox477::unc-119(+)) II oxSi883[Phsp-16.41::TEV(protease) unc-119(+)] II ; oxSi890[Pdpy-30::HA:tagRFP:Mm_SGIP1(97-184) unc-119(+)] IV ; apm-2(ox546[W64X]) X oxSi877[Papm-2::3xFLAG:APM-2:tev-site unc-119(+)] X*

EG8607 *fcho-1(ox477::unc-119(+)) II oxSi883[Phsp-16.41::TEV(protease) unc-119(+)] II ; oxSi891[Pdpy-30::HA:tagRFP:Hs_FCHo1(305-402) unc-119(+)] IV ; apm-2(ox546[W64X]) X oxSi877[Papm-2::3xFLAG:APM-2:tev-site unc-119(+)] X*

Figure 7-figure supplement 1D

Cellular localization of APA

EG8807 *fcho-1(ox477::unc-119(+)) II oxSi254[Pdpy-30::APA-2::GFP unc-119(+)] II ; oxSi890[Pdpy-30::HA:tagRFP:Mm_SGIP1(97-184) unc-119(+)] IV*

EG8808 *fcho-1(ox477::unc-119(+)) II oxSi254[Pdpy-30::APA-2::GFP unc-119(+)] II ; oxSi890[Pdpy-30::HA:tagRFP:Mm_SGIP1(97-184) unc-119(+)] IV ; apm-2(ox546[W64X]) X*

Figure 8A

Cargo assay

EG8578 fcho(+) and EG8579 fcho(-), see Figure 5 cargo assay

EG8809 *oxSi484[Pvha-6::GFP:CD4:YASV unc-119(+)] II ; apm-2(ox546[W64X]) X oxSi882[Papm-2::HA:APM-2(R440S):tev-site X*

EG8810 *oxSi484[Pvha-6::GFP:CD4:YASV unc-119(+)] II ; apm-2(ox546[W64X]) X oxSi880[Papm-2::HA:APM-2(E306K):tev-site unc-119(+)] X*

EG8811 *oxSi484[Pvha-6::GFP:CD4:YASV unc-119(+)] II ; apm-2(ox546[W64X]) X oxSi878[Papm-2::HA:APM-2(T160A):tev-site unc-119(+)] X*

EG8812 *oxSi484[Pvha-6::GFP:CD4:YASV unc-119(+)] II ; apm-2(ox546[W64X]) X oxSi879[Papm-2::HA:APM-2(T160E):tev-site unc-119(+)] X*

EG8813 *oxSi484[Pvha-6::GFP:CD4:YASV unc-119(+)] II ; apm-2(ox546[W64X]) X oxSi881[Papm-2::HA:APM-2(K411E):tev-site unc-119(+)] X*

Figure 8B,C

AP2 FRAP and clustering

EG8814 *oxSi254[Pdpy-30::APA-2::GFP unc-119(+)] II ; apm-2(ox546[W64X]) X oxSi876[Papm-2::HA:APM-2:tev-site unc-119(+)] X*

EG8815 *fcho-1(ox477::unc-119(+)) II oxSi254[Pdpy-30::APA-2::GFP unc-119(+)] II ; apm-2(ox546[W64X]) X oxSi876[Papm-2::HA:APM-2:tev-site unc-119(+)] X*

EG8816 *oxSi254[Pdpy-30::APA-2::GFP unc-119(+)] II ; apm-2(ox546[W64X]) X oxSi882[Papm-2::HA:APM-2(R440S):tev-site X*

EG8817 *oxSi254[Pdpy-30::APA-2::GFP unc-119(+)] II ; apm-2(ox546[W64X]) X oxSi880[Papm-2::HA:APM-2(E306K):tev-site unc-119(+)] X*

EG8818 *oxSi254[Pdpy-30::APA-2::GFP unc-119(+)] II ; apm-2(ox546[W64X]) X oxSi878[Papm-2::HA:APM-2(T160A):tev-site unc-119(+)] X*

EG8819 *oxSi254[Pdpy-30::APA-2::GFP unc-119(+)] II ; apm-2(ox546[W64X]) X oxSi879[Papm-2::HA:APM-2(T160E):tev-site unc-119(+)] X*

EG8820 *oxSi254[Pdpy-30::APA-2::GFP unc-119(+)] II ; apm-2(ox546[W64X]) X oxSi881[Papm-2::HA:APM-2(K411E):tev-site unc-119(+)] X*

**(B) Plasmids**

pGH388 [1-2]HA_TagRFP_wormFCHO-1(536-968)

pGH389 [1-2]HA_TagRFP_wormFCHO-1(1-968)

pGH393 Pdpy-30_HA_TagRFP_wormFCHO-1(536-968)cxTi10882MosSCI

pGH394 Pdpy-30_HA_TagRFP_wormFCHO-1(1-968)cxTi10882MosSCI

pGH424 mouse beta trunk (1-591)_6xHis / mouse mu2 (1-435) hemicomplex

pGH441 6xHis_mouse mu2 linker + mu domain (122-435)

pGH442 [1-2]apm-2

pGH443 [1-2]apm-2(minigene)

pGH444 [1-2]apm-2(minigene+tevsite)

pGH445 [1-2]apm-2(minigene+HA+linker+tevsite)

pGH446 [1-2]apm-2(minigene+3xFlag+tevsite)

pGH447 apm-2(HA+tevsite) ttTi14024 MosSCI

pGH448 apm-2(3xFlag+tevsite) ttTi14024 MosSCI

pGH449 [1-2]apm-2(E306K+HA+tevsite)

pGH450 [1-2]apm-2(K411E+HA+tevsite)

pGH451 [1-2]apm-2(R440S+HA+tevsite)

pGH452 [1-2]apm-2(T160A+HA+tevsite)

pGH453 [1-2]apm-2(T160E+HA+tevsite)

pGH454 apm-2(E306K+HA+tevsite) ttTi14024 MosSCI

pGH455 apm-2(K411E+HA+tevsite) ttTi14024 MosSCI

pGH456 apm-2(R440S+HA+tevsite) ttTi14024 MosSCI

pGH457 apm-2(T160A+HA+tevsite) ttTi14024 MosSCI

pGH458 apm-2(T160E+HA+tevsite) ttTi14024 MosSCI

pGH459 Phsp16_41_TEV_u54UTR

pGH460 Phsp16_41_TEV_u54UTR ttTi5605MosSCI

pGH461 [4-1]Papm-2

pGH462 [2-3]apm-2 3'UTR

pGH463 HaloTag_wormFCHO-1(454-565) pcDNA5frt

pGH464 HaloTag_mouseFCHo2(306-394) pcDNA5frt

pGH465 HaloTag_mouseFCHo1(304-393) pcDNA5frt

pGH466 HaloTag_mouseSGIP1(97-184) pcDNA5frt

pGH467 HaloTag_mouseFCHo2(1-809) pcDNA5frt

pGH468 HaloTag_mouseFCHo2(263-809) pcDNA5frt

pGH469 HaloTag_mouseSGIP1(1-854) pcDNA5frt

pGH470 HaloTag_mouseFCHo1(267-609) pcDNA5frt

pGH471 HaloTag_humanFCHo1(305-402) pcDNA5frt

pGH472 HaloTag_humanFCHo1(267-609) pcDNA5frt

pGH473 HaloTag_fishFCHo1(295-390) pcDNA5frt

pGH474 HaloTag_fishFCHo1(260-609) pcDNA5frt

pGH475 [1-2]HA_TagRFP_wormFCHO-1(288-968)

pGH476 [1-2]HA_TagRFP_wormFCHO-1(1-687)

pGH477 [1-2]HA_TagRFP_wormFCHO-1(1-286)

pGH478 [1-2]HA_TagRFP_wormFCHO-1(454-565)

pGH479 [1-2]HA_TagRFP_mouseFCHo2(306-394)

pGH480 [1-2]HA_TagRFP_mouseFCHo1(304-393)

pGH481 [1-2]HA_TagRFP_mouseSGIP1(97-184)

pGH482 [1-2]HA_TagRFP_humanFCHo1(305-402)

pGH483 Pdpy-30_HA_TagRFP_wormFCHO-1(288-968) cxTi10816 MosSCI

pGH484 Pdpy-30_HA_TagRFP_wormFCHO-1(1-687) cxTi10816 MosSCI

pGH485 Pdpy-30_HA_TagRFP_wormFCHO-1((1-286) cxTi10816 MosSCI

pGH486 Pdpy-30_HA_TagRFP_wormFCHO-1(454-565) cxTi10816 MosSCI

pGH487 Pdpy-30_HA_TagRFP_mouseFCHo2(306-394) cxTi10816 MosSCI

pGH488 Pdpy-30_HA_TagRFP_mouseFCHo1(304-393) cxTi10816 MosSCI

pGH489 Pdpy-30_HA_TagRFP_mouseSGIP1(97-184) cxTi10816 MosSCI

pGH490 Pdpy-30_HA_TagRFP_humanFCHo1(305-402) cxTi10816 MosSCI

pGH491 6xHis_mouse beta linker + appendage domain (592-951)

pGH492 6xHis_mouse alpha linker + appendage domain (622-938)

pGH493 6xHis_HaloTag_mouse SGIP APA domain (97-184)

pGH494 6xHis_HaloTag

pMG114 [1-2]GFP-CD4-YASV

pMG122 Pvha-6_GFP_CD4_YASV ttTi5605 MosSCI

pMT2 [2-3]fcho-1 right targeting arm, for MosDEL

pMT5 [4-1]fcho-1 left targeting arm, for MosDEL

pMT7 fcho-1::unc-119(+) MosDEL targeting

pRL8 [1-2]C. briggsae unc-119(+)

[2-3]unc-54 3’UTR

[4-1]Pdpy-30

[4-1]Pvha-6

mouse alpha trunk (1-621)_GST / mouse sigma2 (1-143) hemicomplex

**(C) Oligonucleotides**

jowls screen and *fcho-1* suppressor screen

oGH408 GCATTTTTCACATTTTCTAACATTTTTTCTGTTGAAAAG

oGH409 GCACATTTTAAGTCTGTAAAAGTGAAAACCCA

oGH411 TCCTATGCTCAGTCAGTGTATGAGC

oGH412 GCTTTTGGAGCATTTTGTTTTCTAATTTTGAATGA

oGH413 AAGTTTTATACCAAGTTTAGAACATGGATTCGG

oGH414 CGGTTCTGCATGCAGTTGTCTG

oGH415 CCAAAAAAATGTATCTGAATAAGTAAAGCAAAGTGATTC

oGH416 GTCTTAACCAAAGAGCAACAACAATACCT

oGH417 ACTATAACTTTTGATTGTTTTGTCAACAGCTAGC

oGH418 CCACTTTTTCTAATATTTCAAACTTGTGCTCGA

oGH419 TGTAAAAGTGGAGAGATGGCACGG

oGH420 ACGGCGAGTTCAGACACTTTTGG

oGH421 CGATTTTAACCACTGAAATGTCACTTTTCTTCG

oGH423 CGGTCGTTGATATTCGCGGAGATTTATG

oGH424 AAAAACGTTTATTAGCTAAAATTTTTATTTAAATTTTTTAAAAAATTTACCG

oGH425 CAGACCAATTTCTTCGTTTTTTAATTGATTTTTTCC

oGH428 CTTTTTAAATGCAAACGCCAAAAATAAAGCC

oGH429 CAGATTTCGCGAGAATTATGCTATATTTACACAG

oGH430 GTTTTGCAAAGATATTTTAATGAAGTTTGGCTCA

oGH432 AAATTAATTGTTTCTACAGAGTGTTTCAATGTTTGAAC

oGH433 TTCGCAATTAATATTAAAAATTCAAATTTTCTCAAAAACACAG

oGH441 GCTCCAATTTCCTTGAAACCTCG

oGH442 CCTTGAAAGCTTTTTTTAAGTTTTTTAGGTG

oGH443 GATTTTTCAAAATTTTTAACATCGAAACTCCC

oGH444 GCCCGATTTTACAGGAACTCC

oGH445 CTAAAATTCTAAACTACAAAATAATAATAAAAATATC

oGH446 TGCAATTTTTACAGGTCAGG

oGH447 CTCGGAAATTCAAATTATACATCAAAAATTATCAC

oGH448 GAAATTCAGAATTATTTAGGGGAAAAGGC

oGH450 CATTTTGATTTAGCAGACCCATTGTAA

oGH451 GTTTTTGAACCTATTTTTGTAGTTTGTAGTTG

oGH452 CCATTCATATTTTGTCTCAGGAGAATAC

*fcho-1* MosDEL

oMT1 GGGGACAGCTTTCTTGTACAAAGTGGAGTCTAGCAGACCCAAACTTGTG

oMT2 GGGGACAACTTTGTATAATAAAGTTGATCCTTGCTTTCAGCGATCC

oMT3 GGGGACAACTTTGTATAGAAAAGTTGGACCCATAGAATTGGGTCTGCT

oMT4 GGGGACTGCTTTTTTGTACAAACTTGCACTTCTTGCAGCCGTATCTGT

TEV assay transgenes

oGH519 GGGGACAACTTTGTATAATAAAGTTGGTTTTATACCAAGTTTAGAACATGGATTCGG

oGH616 CAGCTTTCTTGTACAAAGTTGGCATTATAAGAAAGCATTGCTT

oGH617 CATTTCGGCGGTACTTAATACCCTCCCGACGCCATCCAATTTGG

oGH618 TATTAAGTACCGCCGAAATGAGCTC

oGH619 TTTCATTCAAGTTTTAATCCTATGCTCAGTCAGTGT

oGH620 GGATTAAAACTTGAATGAAAAAGTTCTCACAGGC

oGH621 AACTTTGTACAAGAAAGCTGCTAGCATCTGGTTTCATACAGTCC

oGH634 GGGGACAAGTTTGTACAAAAAAGCAGGCTCAAAAATGATTGGTGGATTGTTCGTTTACA

oGH635 GGGGACCACTTTGTACAAGAAAGCTGGGTCTAGCATCTGGTTTCATACAGTCCC

oGH699 GGGGACCACTTTGTACAAGAAAGCTGGGTCTAGCATCTGGTTTCATACAGTCC

oGH737 GGGGACAAGTTTGTACAAAAAAGCAGGCTCAAAAATGTACCCATACGACGTCCCT

oGH751 GTGAGCTCTGGTACCCTCTAG

oGH752 GCGCGATGCATTCGAAGATCTG

oGH753 ATGTACCCATACGACGTCCCTGACTACGCTATGATTGGTGGATTG

oGH756 GAACCTCTACTTCCAAGGATCCAGCCGTGCCGCAGTG

oGH757 ATCCTTGGAAGTAGAGGTTCTCTCTGCAAACAATAAAGTATCAGTTTCC

oGH785 GGGGACAACTTTGTATAGAAAAGTTGCTCTATTTATCTCATAGATTTTGTTCTGCGTTG

oGH786 GGGGACTGCTTTTTTGTACAAACTTGCCCCGAGTTTGCTGAAATGTAT

oGH797 GGGGACAGCTTTCTTGTACAAAGTGGAACTTCACCCAACCCTTTCTATCTTC

oGH806 TAGAGGGTACCAGAGCTCACGATCACCAAAAACGGAACGTTG

oGH807 GATCTTCGAATGCATCGCGCAAACAGTTATGTTTGGTATATTGGGAATG

oGH814 CATGATCTTTATAATCACCGTCATGGTCTTTGTAGTCCATTTTTGAGCCTGCTTTTTTGT

oGH815 TAAAGATCATGACATCGATTACAAGGATGACGATGACAAGATGATTGGTGGATTGTTCGT

oGH929 CTCAAGTGGCTGGCCAAATTGGATGGCGTC

oGH930 ATTTGGCCAGCCACTTGAGACGTGATTTGTGACT

oGH931 CTCAAGTGGAAGGCCAAATTGGATGGCGT

oGH932 ATTTGGCCTTCCACTTGAGACGTGATTTGTGACT

oGH937 ACAAGATGAAAGTTAAGGTATTTCACTTGTCACTTCAAATATT

oGH938 ACCTTAACTTTCATCTTGTTACGAGACACTTCACG

oGH943 GCTACTTGGAGGTGTTTGAGCCAAAACTGAACT

oGH944 TCAAACACCTCCAAGTAGCGAACTTTGAGTCCAG

oGH947 ATGAAACCAGCTGCTAGACCCAGCTTTCTTG

oGH948 GTCTAGCAGCTGGTTTCATACAGTCCCGATCTTC

*fcho-1* structure/function

oCF590 AGTACTAGCGGTGGCAGT GGAGGTACCGGCGGAAGC AGTGTGTCTAAGGGCG

oCF591 ACCAGTGCCTCCACTACC GCCCGTTCCTCCTGTGCCA CCTCCGGAATTAAGTT

oGH323 GGGGACAAGTTTGTACAAAAAAGCAGGCTCAAAAATGACGGCTGAGGGTC

oGH324 GGGGACCACTTTGTACAAGAAAGCTGGGTCTATTTCCGCAGCTCGG

oGH350 CCACTGCCACCGCTAGTACTCATTTTTGAGCCTGCTTTTTTGTAC

oGH352 GCGGTAGTGGAGGCACTGGTACGGCTGAGGGTCTGC

oGH372 GTACCCATACGACGTCCCTGACTACGCTCGTAC

oGH373 GAGCGTAGTCAGGGACGTCGTATGGGTACGTAC

oGH649 TAGACCCAGCTTTCTTGTACAAAGTTGGCATTATAAG

oGH653 ACTCGACGGGCCGCAACCCATTTTTGAGCCTGCTTTTTTGTACAAAGTTGGCATTATAAA

oGH657 GCGGCCCGTCGAGTGGACGACAAAATCCGTCGACTAGTACTAGCGGTGGCAGTGGAGGTA

oGH781 ACCAGTGCCTCCACTACCGCCCGT

oGH793 GCGGTAGTGGAGGCACTGGTGGCTCGGCGCCGGCTCAC

oGH794 GTACAAGAAAGCTGGGTCTATGTTCCGCCAATCGTCCAGGGATC

oGH808 GCGGTAGTGGAGGCACTGGTGAATGTCCTGATGCAGATTCGC

oGH809 GTACAAGAAAGCTGGGTCTAACTGTGTCTAGATACTGCTGGG

oGH810 GCGGTAGTGGAGGCACTGGTGATTTCCTGGAGTCTGACTCAGG

oGH811 GTACAAGAAAGCTGGGTCTACATGGTGCCCCCTGGG

oGH812 GCGGTAGTGGAGGCACTGGTGCAGAAATCGATTGGGAAAGATA

oGH813 GTACAAGAAAGCTGGGTCTAACTTTTCCTCACAGGCGAAG

oGH1035 GCGGTAGTGGAGGCACTGGTGCAGCTGTAGATTTCCTGGAGC

oGH1036 GTACAAGAAAGCTGGGTCTATGAAGAATGGCGTTTCATGG

MudPIT and tissue culture

oGH828 ACTTTCAGAGCGATAACTTAATTAACGGCTCGGCGCCGGCTCAC

oGH829 CGAGGCTGATCAGCGGGTTTAAACCTATGTTCCGCCAATCGTCCAGGGATC

oGH830 ACTTTCAGAGCGATAACTTAATTAACGAATGTCCTGATGCAGATTCGC

oGH831 CGAGGCTGATCAGCGGGTTTAAACCTAACTGTGTCTAGATACTGCTGGG

oGH832 ACTTTCAGAGCGATAACTTAATTAACGATTTCCTGGAGTCTGACTCAGG

oGH833 CGAGGCTGATCAGCGGGTTTAAACCTACATGGTGCCCCCTGGG

oGH834 ACTTTCAGAGCGATAACTTAATTAACGCAGAAATCGATTGGGAAAGATA

oGH835 CGAGGCTGATCAGCGGGTTTAAACCTAACTTTTCCTCACAGGCGAAG

oGH886 ACTTTCAGAGCGATAACTTAATTAACATGGTCATGGCGCATTTCGTG

oGH887 CGAGGCTGATCAGCGGGTTTAAACTCAACAATCTGCCAAGTATCGTCC

oGH890 ACTTTCAGAGCGATAACTTAATTAACATGATGGAAGGACTGAAAAAACGTAC

oGH891 CGAGGCTGATCAGCGGGTTTAAACTTAGTTATCTGCCAAGTACTTTCCTG

oGH892 ACTTTCAGAGCGATAACTTAATTAACGAAAGACCTGGTCTTATTGAATTTGAAGAG

oGH1019 ACTTTCAGAGCGATAACTTAATTAACGACTTCGATGCATACAGCTCAG

oGH1020 CGAGGCTGATCAGCGGGTTTAAACCTAGGCGTCCTGAGATCCCAG

oGH1021 ACTTTCAGAGCGATAACTTAATTAACGACTTCGAGGCATACAGTGC

oGH1022 CGAGGCTGATCAGCGGGTTTAAACCTAGTGTCCTGTCTGGGATAAGAAGC

oGH1023 ACTTTCAGAGCGATAACTTAATTAACGGATTTGAGGAATATCTGTCCTCG

oGH1024 CGAGGCTGATCAGCGGGTTTAAACCTAACTTCCAATCATAGTAATAGCAAAAGGAT

oGH1039 ACTTTCAGAGCGATAACTTAATTAACGCAGCTGTAGATTTCCTGGAG

oGH1040 CGAGGCTGATCAGCGGGTTTAAACCTATGAAGAATGGCGTTTCATGGT

oGH1041 ACTTTCAGAGCGATAACTTAATTAACTCAACGGATTCAGCTGTTGC

oGH1042 CGAGGCTGATCAGCGGGTTTAAACCTATGCGCAAACCCTCCTGTT

Bacterial expression

oGH332 CATGGTATATCTCCTTATTAAAGTTAAACAAAATTATTTC

oGH336 GCCATCACCATCATCACCACTAATGCTTAAGTCGAACAGAAAGTAATCG

oGH338 CATATGTATATCTCCTTCTTATACTTAACTAATATACTAAGATG

oGH339 TAATTAACCTAGGCTGCTGCCA

oGH368 TAATAAGGAGATATACCATGACTGACTCCAAGTACTTCACAAC

oGH370 AAGAAGGAGATATACATATGATCGGAGGCTTATTCATCTATAATCAC

oGH371 GCAGCAGCCTAGGTTAATTAGCAGCGGGTTTCATAAATGC

oGH571 GTGGTGATGATGGTGATGCATGGTATATCTCCTTATTAAAGTTAAACAAAATTATTTC

oGH676 GTGGTGATGATGGTGATGGCTGCTGCCTTTGCGATGAATCCCATGACTC

oGH853 TAATTAACCTAGGCTGCTGCCACCG

oGH861 GCAGCAGCCTAGGTTAATTAACTTTTCCTCACAGGCGAAG

oGH921 TGCATCACCATCATCACCACCAGAACTCAGAGACAGGTGCACTG

oGH1161 TGCATCACCATCATCACCACCACTTGCCAATTCATCATGGGAG

oGH1162 GCAGCAGCCTAGGTTAATTAGTTTTTCAAAATGCTGTCGTAGACC

oGH1163 TGCATCACCATCATCACCACAGCACAGTGACTGACCTGG

oGH1164 GCAGCAGCCTAGGTTAATTAGAACTGTTCCGACAGCAATTCAC

oGH1165 GTGGTGATGATGGTGATGCATG

oGH1166 TGCATCACCATCATCACCACGCAGAAATCGGTACTGGCTTTC

oGH1167 ATTAAAACAGATGCACGACGGTTATCGCTCTGAAAGTACAGATCC

oGH1169 TCGTGCATCTGTTTTAATTAACGCAGAAATCGATTGGGAAAGATA

oGH1171 TAATTAAAACAGATGCACGACGGTTATCGCTCTGAAAGTACAGAT

oGH1170 GTGCATCTGTTTTAATTAACTAATTAACCTAGGCTGCTGCCACCG
